# Recombinant Rotaviruses Rescued by Reverse Genetics Reveal the Role of NSP5 Hyperphosphorylation in the Assembly of Viral Factories

**DOI:** 10.1128/JVI.01110-19

**Published:** 2019-12-12

**Authors:** Guido Papa, Luca Venditti, Francesca Arnoldi, Elisabeth M. Schraner, Christiaan Potgieter, Alexander Borodavka, Catherine Eichwald, Oscar R. Burrone

**Affiliations:** a International Centre for Genetic Engineering and Biotechnology (ICGEB), Trieste, Italy; b Department of Medicine, Surgery and Health Sciences, University of Trieste, Trieste, Italy; c Institute of Virology, University of Zurich, Zurich, Switzerland; d Deltamune (Pty) Ltd., Lyttelton, Centurion, South Africa; e Department of Biochemistry, Focus Area Human Metabolomics, North-West University, Potchefstroom, South Africa; f Astbury Centre for Structural Molecular Biology, School of Molecular and Cellular Biology, University of Leeds, Leeds, United Kingdom; Icahn School of Medicine at Mount Sinai

**Keywords:** NSP5, reverse genetics, rotavirus, viral factories, viroplasms, protein phosphorylation, recombinant viruses

## Abstract

Rotavirus (RV) replicates in round-shaped cytoplasmic viral factories, although how they assemble remains unknown. During RV infection, NSP5 undergoes hyperphosphorylation, which is primed by the phosphorylation of a single serine residue. The role of this posttranslational modification in the formation of viroplasms and its impact on virus replication remain obscure. Here, we investigated the role of NSP5 during RV infection by taking advantage of a modified fully tractable reverse-genetics system. A *trans*-complementing cell line stably producing NSP5 was used to generate and characterize several recombinant rotaviruses (rRVs) with mutations in NSP5. We demonstrate that an rRV lacking NSP5 was completely unable to assemble viroplasms and to replicate, confirming its pivotal role in rotavirus replication. A number of mutants with impaired NSP5 phosphorylation were generated to further interrogate the function of this posttranslational modification in the assembly of replication-competent viroplasms. We showed that the rRV mutant strains exhibited impaired viral replication and the ability to assemble round-shaped viroplasms in MA104 cells. Furthermore, we investigated the mechanism of NSP5 hyperphosphorylation during RV infection using NSP5 phosphorylation-negative rRV strains, as well as MA104-derived stable transfectant cell lines expressing either wild-type NSP5 or selected NSP5 deletion mutants. Our results indicate that NSP5 hyperphosphorylation is a crucial step for the assembly of round-shaped viroplasms, highlighting the key role of the C-terminal tail of NSP5 in the formation of replication-competent viral factories. Such a complex NSP5 phosphorylation cascade may serve as a paradigm for the assembly of functional viral factories in other RNA viruses.

**IMPORTANCE** The rotavirus (RV) double-stranded RNA genome is replicated and packaged into virus progeny in cytoplasmic structures termed viroplasms. The nonstructural protein NSP5, which undergoes a complex hyperphosphorylation process during RV infection, is required for the formation of these virus-induced organelles. However, its roles in viroplasm formation and RV replication have never been directly assessed due to the lack of a fully tractable reverse-genetics (RG) system for rotaviruses. Here, we show a novel application of a recently developed RG system by establishing a stable *trans*-complementing NSP5-producing cell line required to rescue rotaviruses with mutations in NSP5. This approach allowed us to provide the first direct evidence of the pivotal role of this protein during RV replication. Furthermore, using recombinant RV mutants, we shed light on the molecular mechanism of NSP5 hyperphosphorylation during infection and its involvement in the assembly and maturation of replication-competent viroplasms.

## Abstract (precis)

The rotavirus (RV) double-stranded RNA genome is replicated and packaged into virus progeny in cytoplasmic structures termed viroplasms. The nonstructural protein NSP5, which undergoes a complex hyperphosphorylation process during RV infection, is required for the formation of these virus-induced organelles. However, its roles in viroplasm formation and RV replication have never been directly assessed due to the lack of a fully tractable reverse-genetics (RG) system for rotaviruses. Here, we show a novel application of a recently developed RG system by establishing a stable *trans*-complementing NSP5-producing cell line required to rescue rotaviruses with mutations in NSP5. This approach allowed us to provide the first direct evidence of the pivotal role of this protein during RV replication. Furthermore, using recombinant RV mutants, we shed light on the molecular mechanism of NSP5 hyperphosphorylation during infection and its involvement in the assembly and maturation of replication-competent viroplasms.

## INTRODUCTION

Rotavirus (RV) is the most common cause of viral gastroenteritis in young children and infants worldwide (1, 2). It is a nonenveloped RNA virus with a genome composed of 11 segments of double-stranded RNA (dsRNA), which replicates in cytoplasmic structures composed primarily of viral proteins (3–5). During infection, the first steps of viral morphogenesis and genome replication occur within cytoplasmic viral replication factories known as viroplasms (3, 5–7).

The assembly of viroplasms requires coexpression of at least two nonstructural proteins, NSP5 and NSP2 (8, 9); however, how these virus-induced organelles are formed remains unknown.

Other viral proteins also found in viroplasms include RNA-dependent RNA polymerase (RdRp) VP1, the main inner-core protein VP2, guanyltransferase/methylase VP3, and the middle layer (inner capsid) protein VP6 (10, 11). Biochemical evidence suggests that viroplasms are essential for RV replication, since virus production is highly impaired upon silencing of either NSP2 or NSP5 (12–15).

Rotavirus NSP5, encoded by genome segment 11 (gs11), is a small serine (Ser)- and threonine (Thr)-rich nonstructural protein that undergoes multiple posttranslational modifications in virus-infected cells, including O-linked glycosylation (16), N-acetylation (17), SUMOylation (18), and, crucially, hyperphosphorylation, which involves several distinct Ser residues (19, 20). NSP5 hyperphosphorylation is a complex process which gives rise to multiple phosphorylation states ranging from the most abundant 28-kDa phosphoisoform up to the hyperphosphorylated 32- to 34-kDa states (19, 20). All of these forms have been found to be more stable in viroplasms, while chemical disruption of viroplasms results in NSP5 dephosphorylation (21). The mechanism of NSP5 phosphorylation is not yet wholly understood, but it involves interactions with other viral proteins. When expressed alone in noninfected cells, NSP5 is not phosphorylated, while coexpression with NSP2 or VP2 results in NSP5 hyperphosphorylation and formation of viroplasm-like structures (VLS) (8, 22, 23). NSP5 hyperphosphorylation involves the phosphorylation of serine 67 (Ser67) by casein kinase 1α (CK1α) to initiate the phosphorylation cascade (24, 25), and it is considered to be essential for the assembly of viroplasms (26).

Although the structure of NSP5 remains unknown, it readily forms higher-molecular-weight oligomeric species in solution, potentially providing a larger interface for interacting with multiple components of viroplasms (27).

In addition, the C-terminal region (a “tail” including amino acids [aa] 180 to 198) is required for NSP5 decamerization *in vitro* (27) and VLS formation *in vivo* (7).

However, due to the lack of a fully tractable reverse-genetics (RG) system for RVs until recently, previous studies on NSP5 have been carried out using mutants expressed in the absence of a complete set of viral proteins. Here, we took advantage of the novel plasmid-only-based, helper virus-free RG systems for rotaviruses (28, 29) to gain new insights into the mechanisms of NSP5 hyperphosphorylation and its role in viroplasm assembly and virus replication during viral infection.

To achieve this, we generated and characterized several viable recombinant rotaviruses (rRVs) with mutations in NSP5. Using these mutants, we show the role of NSP5 hyperphosphorylation for viroplasm assembly and in genome replication. These studies shed light on a complex hierarchical mechanism of NSP5 hyperphosphorylation during rotaviral infection.

(This article was submitted to an online preprint archive [30].)

## RESULTS

### Generation of gs11 mutated recombinant RVs.

Recombinant rotaviruses (simian rotavirus A strain SA11) carrying different mutations in the NSP5 coding region of gs11 were obtained using a recently developed reverse-genetics protocol (29). Two essential additional modifications were introduced to *trans*-complement the potential loss of NSP5 function in the rRV mutants: (i) an additional plasmid, pcDNA3-NSP5 (T7 and cytomegalovirus [CMV] promoter driven), encoding the open reading frame (ORF) of wild-type (wt) NSP5 (i.e., without gs11 5′ and 3′ untranslated regions [UTRs]), was included in the transfection step of BHK-T7 cells, and (ii) each rescued rRV was amplified in a stable transfectant cell line, MA104-NSP5 (MA-NSP5), supplying the wt NSP5 in *trans*. Crucially, we have successfully established a novel MA-NSP5 cell line to support the replication of NSP5-deficient recombinant viruses to enable their further in-depth characterization. Both steps i and ii were absolutely required for rescuing the NSP5/KO virus, confirming the essential role of NSP5 for RV replication. These mutants, as well as additional stable cell lines generated for this study as described in Materials and Methods, are summarized in Table 1.

**TABLE 1:**
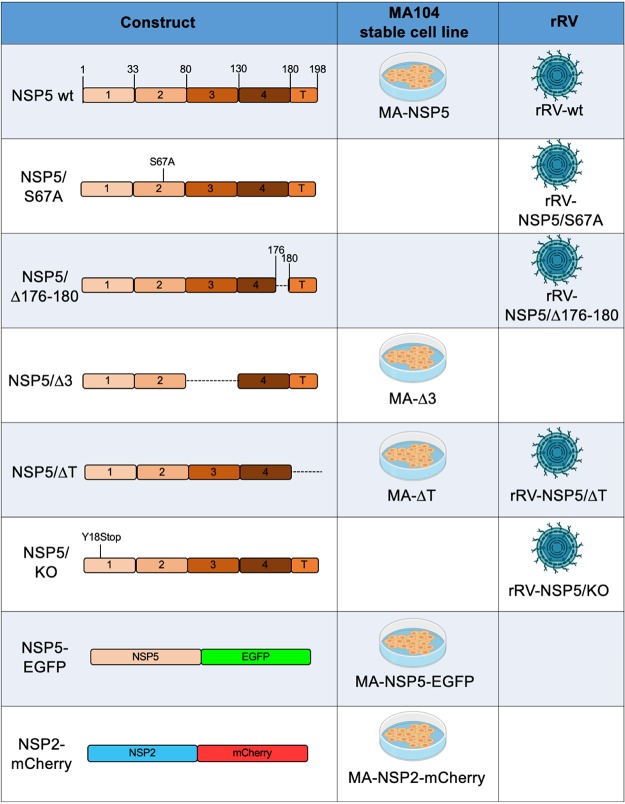
Schematic representation of NSP5 and NSP2 mutants or fusion proteins used to generate rRV and/or stable MA104 transfectant cell lines used in this study^*a*^

a Images in the second and third columns were created using BioRender.

### rRV-NSP5/KO.

NSP5 expression and localization to viroplasms in virus-infected cells have been considered essential for virus replication (12, 13, 25, 31). Previous studies, using small interfering RNA (siRNA) targeting gs11 mRNA, have shown strong impairment of RV replication (12, 13). In order to investigate the effects of point mutations and deletions within the NSP5 gene, we took advantage of the established *trans*-complementing MA104 cell line stably expressing NSP5. In addition, we also made two cell lines expressing NSP2-mCherry and NSP5-enhanced green fluorescent protein (EGFP) fusions, which are rapidly and efficiently recruited into viroplasms upon virus infection (Table 1) (7, 22).

**FIG 1:**
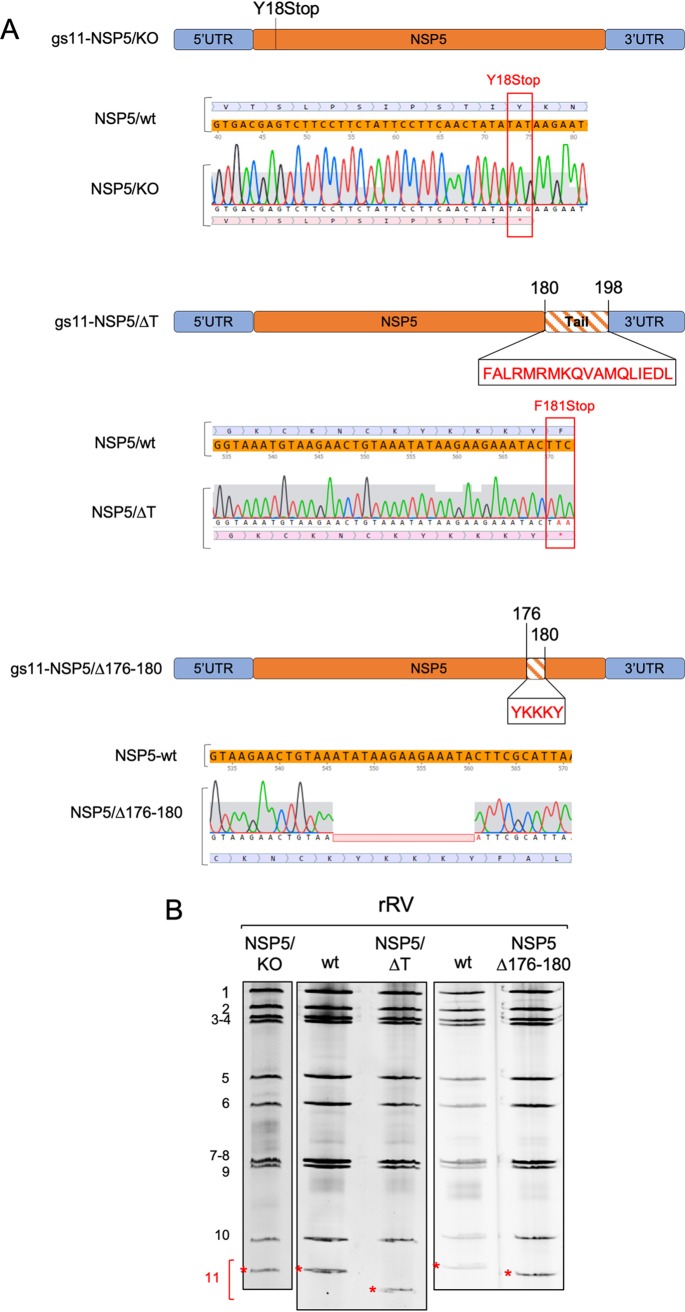
(A) Schematic representation of mutations in gs11 of the corresponding rRV strains. Sequences mutated or deleted in NSP5 are indicated. (B) Profiles of viral dsRNAs of the different rRV strains grown in MA-NSP5 cells. gs11 is indicated by a red asterisk.

**FIG 2:**
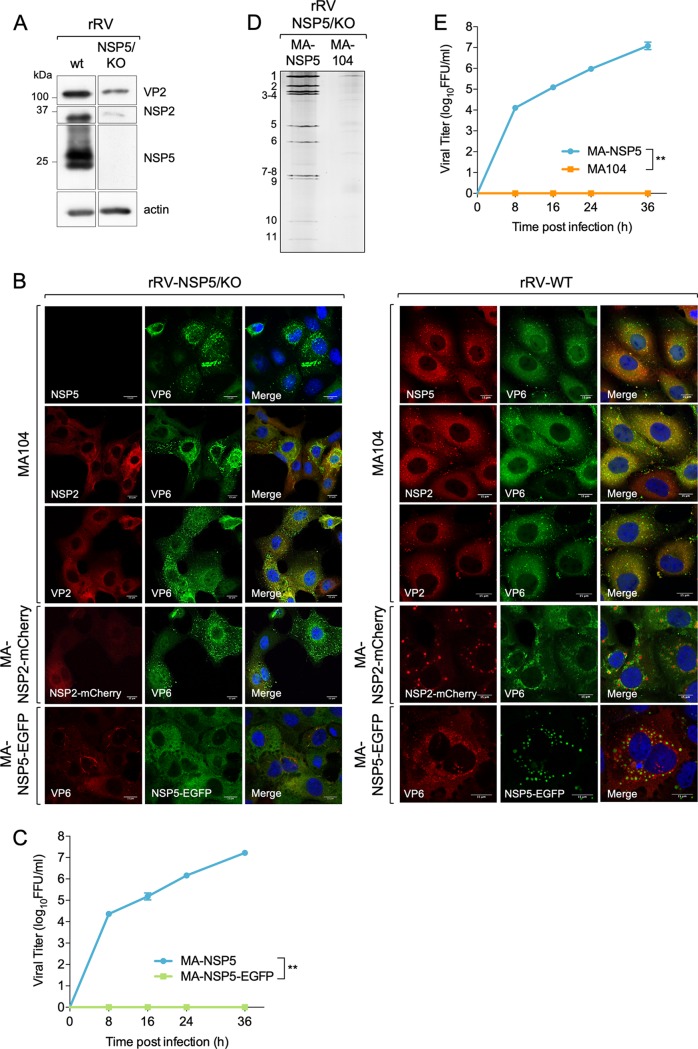
Characterization of rRV-NSP5/KO. (A) Western blot analysis of MA104 cell extracts infected with rRV-wt and rRV-NSP5/KO strains (MOI, 1 FFU/cell). (B) Confocal immunofluorescence microscopy of MA104, MA-NSP2-mCherry, and MA-NSP5-EGFP cells infected with rRV-wt or rRV-NSP5/KO (MOI, 1 FFU/cell), using antibodies for NSP5, NSP2, VP2, and VP6, as indicated. Scale bar, 15 μm. (C) A comparison of the replication kinetics of rRV-NSP5/KO in MA-NSP5 and MA-NSP5-EGFP cells. Data are expressed as the means ± standard deviations (*n* = 3); **, *P* < 0.01 (Student's *t* test). (D) Electrophoretic migration pattern of dsRNAs extracted from the rRV-NSP5/KO strain grown in MA-NSP5 or MA104 cells. Genome segments 1 to 11 are indicated on the left. (E) Replication kinetics of rRV-NSP5/KO in MA-NSP5 and MA104 cells. Data are expressed as the means ± standard deviations (*n* = 3); **, *P* < 0.01 (Student's *t* test).

Here, we provide a direct demonstration of the role of NSP5 in RV replication, using an NSP5 knockout rRV (termed rRV-NSP5/KO) generated by reverse genetics. To rescue the NSP5/KO strain, a stop codon at residue 18Y was introduced (Fig. 1A and B). Analysis of MA104 virus-infected cell extracts confirmed the presence of NSP2 and VP2 but not of NSP5 (Fig. 2A). Moreover, we did not detect viroplasms containing NSP2, VP2, or VP6 in either MA104-infected cells or stable transfectant cell lines expressing the fluorescent fusion protein NSP2-mCherry or NSP5-EGFP (Fig. 2B). Interestingly, this suggests that the NSP5-EGPF fusion protein is not able to *trans*-complement the lack of NSP5, and indeed the rRV-NSP5/KO strain does not replicate in MA-NSP5-EGFP cells (Fig. 2C). Furthermore, both genomic dsRNA synthesis (Fig. 2D) and infectious progeny virus production were completely abrogated in MA104 cells but not in the *trans*-complementing MA-NSP5 cell line (Fig. 2E). Together, these data confirm that NSP5 is essential for RV replication.

### rRVs with impaired NSP5 phosphorylation.

**FIG 3:**
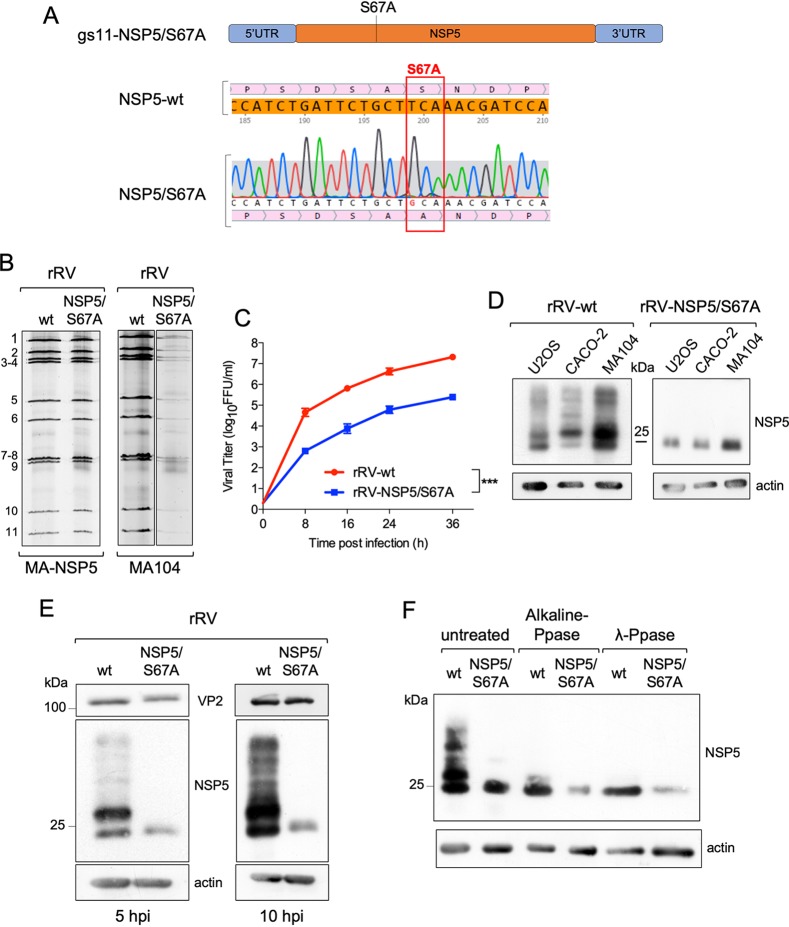
Characterization of rRV-NSP5/S67A. (A) Schematic representation of rRV-NSP5/S67A gs11 and sequence of NSP5-wt and NSP5/S67A mutant (highlighted). (B) Electrophoretic pattern of dsRNA genome segments of rRV-wt and rRV-NSP5/S67A strains grown in MA-NSP5 cells (left panel) and in MA104 wt cells (right panel). (C) Replication kinetics of rRV-wt and rRV-NSP5/S67A in MA104 cells. Data are expressed as the means ± standard deviations (*n* = 3); ***, *P* < 0.001 (Student's *t* test). (D) Western blot analysis of extracts of U2OS, Caco-2, and MA104 cells infected with rRV-wt or rRV-NSP5/S67A strains. (E) Western blot analysis of NSP5 phosphorylation pattern in MA104 cells infected with rRV-wt or rRV-NSP5/S67A (MOI, 1 FFU/cell) at 5 and 10 hpi. (F) Western blot analysis of λ-Ppase and alkaline Ppase treatment of lysates of MA104 cells infected with rRV-wt or rRV-NSP5/S67A. Protein bands corresponding to NSP5 are shown.

To address the role of NSP5 hyperphosphorylation, we then generated a number of rRVs harboring NSP5 mutations previously known to impact NSP5 phosphorylation. We first generated an rRV carrying an S67A mutation (rRV-NSP5/S67A) (Table 1 and Fig. 3A and B). Its replication in wild-type MA104 cells was strongly impaired (Fig. 3B, right panel), resulting in approximately a 100-fold reduction of the infectious progeny virus titer at different time points postinfection (Fig. 3C). Despite the overall reduction of replication fitness, the rRV-NSP5/S67A mutant virus was stable after 10 passages in the wild-type MA104 cells, confirmed by the sequencing of the progeny virus. Consistent with our previous results, the NSP5/S67A mutant was not hyperphosphorylated in all cell lines tested, including MA104, U2OS, and Caco-2 cells (Fig. 3D), further confirming the role of Ser67 in the initiation of NSP5 phosphorylation cascade (24). While the wild-type rRV (rRV-wt) yielded multiple hyperphosphorylated NSP5 isoforms, the NSP5/S67A mutant mostly produced a single, homogeneous form of NSP5 with an apparent mass of 26 kDa that could be detected in the virus-infected cell extracts at 5 or 10 h postinfection (hpi) (Fig. 3E). Enzymatic dephosphorylation with λ-protein phosphatase (λ-Ppase) and alkaline calf intestinal Ppase (CIP), previously used to discriminate phosphorylated from nonphosphorylated NSP5 (19), further corroborated the observed lack of NSP5/S67A phosphorylation (Fig. 3F). Because of the differences in the molecular weight markers used, the NSP5 band with the fastest PAGE mobility has been traditionally described as 26 kDa, and the most abundant one as 28 kDa. Since this nomenclature has been used in many publications, we prefer to maintain it, despite the fact that current PAGE migrations do not correspond to the markers presently used.

**FIG 4:**
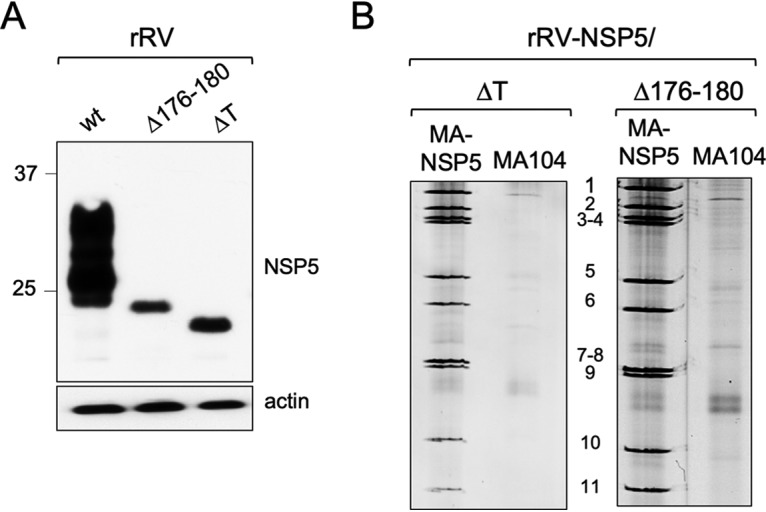
Characterization of rRV-NSP5/ΔT and rRV-NSP5/Δ176-180. (A) Western blot analysis of NSP5 and NSP5 mutants from MA104 cells infected with rRV-wt, rRV-NSP5/ΔT, or rRV-NSP5/Δ176-180 at 5 hpi. (B) Electrophoretic dsRNA migration pattern of rRV-NSP5/ΔT and rRV-NSP5/Δ176-180 grown in MA-NSP5 and MA104 cells.

We then generated two additional rRVs harboring truncated versions of NSP5, one lacking the 18-aa-long C-terminal tail (rRV-NSP5/ΔT) and the second one with a 5-aa deletion (176-YKKKY-180) just upstream of the tail region (rRV-NSP5/Δ176-180) (Table 1 and Fig. 1A and B). The mutant lacking the tail (NSP5/ΔT) has been previously reported as phosphorylation negative when expressed alone in MA104 cells and unable to multimerize (32), while unpublished data from our lab indicated that the NSP5/Δ176-180 mutant also showed no hyperphosphorylation. Despite the presence of Ser67, and the lack of Thr and Ser residues within the deletions, both mutants did not show a classical hyperphosphorylation pattern (Fig. 4A) and failed to replicate, confirmed by the absence of *de novo* synthesis of genomic dsRNA (Fig. 4B).

**FIG 5:**
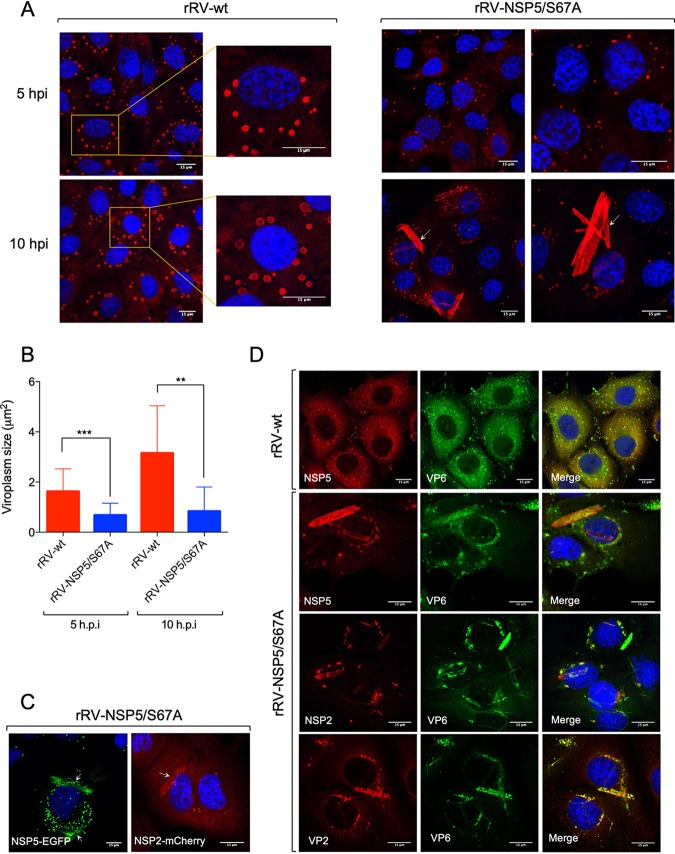

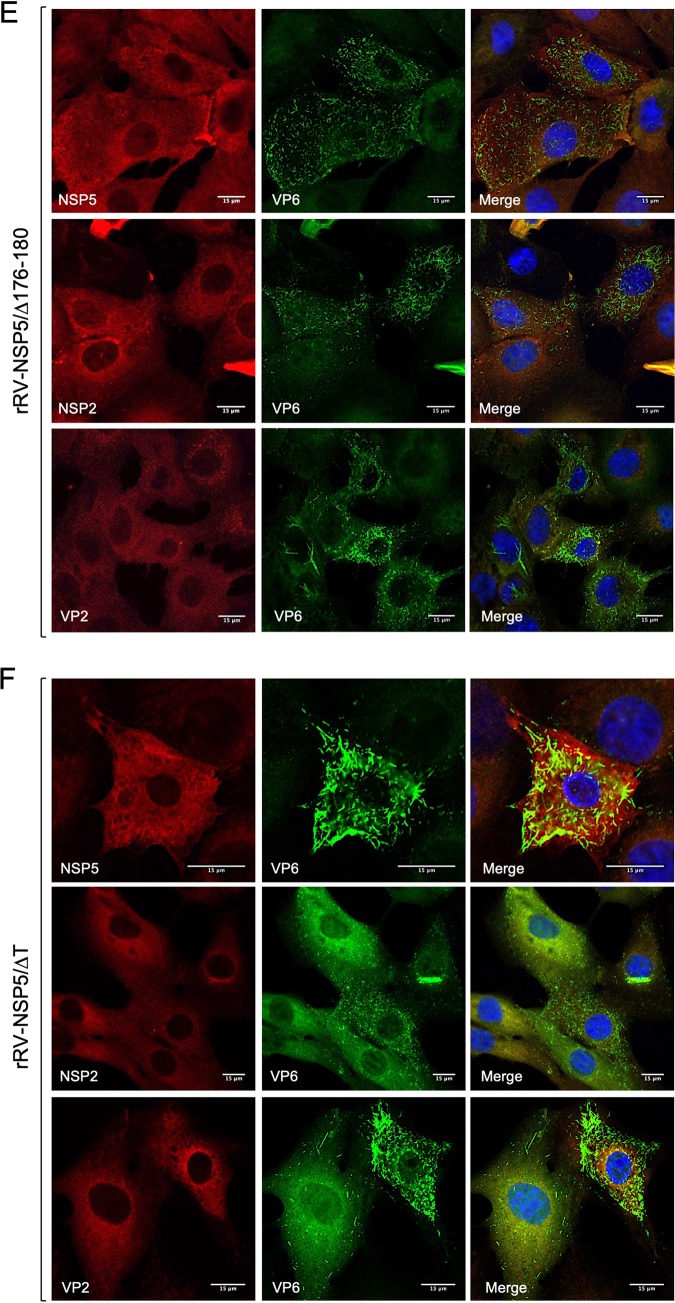
Viroplasm morphology in MA104 cells infected with rRV-NSP5 wt and mutants. (A) Representative confocal immunofluorescence micrographs of MA104 cells infected with rRV-wt and rRV-NSP5/S67A (MOI, 15 FFU/cell) at 5 hpi (upper panels) and 10 hpi (lower panels). Cells were stained with anti-NSP5 and DAPI. (B) Quantitative analysis of viroplasm size (μm^2^) of MA104 cells infected with rRV-wt and rRV-NSP5/S67A at 5 hpi and 10 hpi. ***, *P* < 0.001; **, *P* < 0.01. (C) Confocal immunofluorescence micrographs of MA-NSP5-EGFP and MA-NSP2-mCherry cells infected with rRV-NSP5/S67A (MOI, 15 FFU/cell). (D) Confocal immunofluorescence micrographs of rRV-wt- and rRV-NSP5/S67A-infected MA104 cells (MOI, 15 FFU/cell) stained at 10 hpi with the indicated antibodies. (E and F) Confocal immunofluorescence of MA104 cells infected with rRV-NSP5/Δ176-180 (E) or rRV-NSP5/ΔT (F) and stained with the indicated antibodies and DAPI. Scale bar, 15 μm.

We also investigated viroplasm formation in cells infected with these rRV mutants. During early infection (5 hpi), the rRV-NSP5/S67A mutant produced structures resembling viroplasms that appeared smaller and more heterogeneous in shape than the regular, spherical ones produced during rRV-wt infection (Fig. 5A, upper panel, and Fig. 5B). Remarkably, during late infection (10 to 12 hpi), the rRV-NSP5/S67A mutant produced multiple NSP5-containing aberrant structures, as well as fiber-like structures that became more apparent during late infection (12 hpi) (Fig. 5A, lower panel, and Fig. 5B).

Interestingly, these structures produced by rRV-NSP5/S67A were morphologically similar to those observed during wt RV infection of MA104 cells silenced for CK1α, previously shown to be required for NSP5 phosphorylation (25). NSP2-mCherry and NSP5-EGFP fusion proteins were also recruited to both types of these structures (Fig. 5C), suggesting that the observed lack of phosphorylation does not affect NSP2-NSP5 interactions. Furthermore, the NSP5/S67A mutant and additional viroplasmic proteins, NSP2, VP6, and VP2 (Fig. 5D), were all present in the aberrant structures, suggesting that they could also represent sites of virus replication.

In contrast, no viroplasms containing NSP5, NSP2, or VP2 were observed when MA104 cells were infected with the rRV-NSP5/ΔT and rRV-NSP5/Δ176-180 deletion mutants. Interestingly, some cells infected with these mutants yielded fiber-like structures containing only the VP6 protein (Fig. 5E and F). One possibility is that the appearance of these structures depends on the relative concentration of nonphosphorylated NSP5 in virus-infected cells. Similar VP6 fibers are normally formed when VP6 is overexpressed in cells in the absence of other viral proteins (33, 34).

Taken together, these results confirm the role of NSP5 hyperphosphorylation for controlling the assembly of regular-shaped viroplasms, highlighting the key role of the C-terminal tail in the formation of RV viral factories.

### RNA accumulation in aberrant structures.

**FIG 6:**
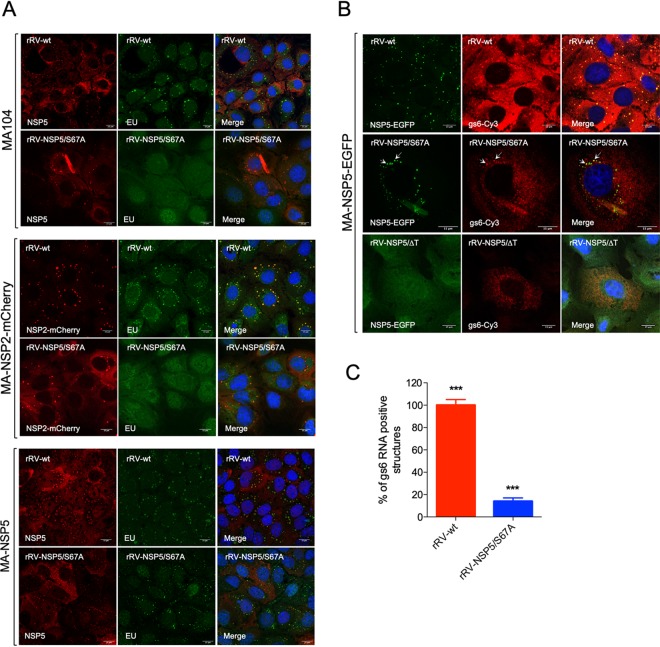
Viral RNA detection in rRV-NSP5/S67A-infected cells. (A) Representative confocal immunofluorescence micrographs of MA104 (upper panel), MA-NSP2-mCherry (middle panel), and MA-NSP5 (lower panel) cells infected with rRV-wt or rRV-NSP5/S67A strains and stained with anti-NSP5 (red in MA104 and MA-NSP5 cells) and 5-EU (green). (B) Single-molecule RNA fluorescence in situ hybridization (smFISH) of MA-NSP5-EGFP cells infected with rRV-wt, rRV-NSP5/S67A, or rRV-NSP5/ΔT strains. Viroplasms detected with NSP5-EGFP (green) and viral RNA (the probe specific for gs6 was Cy3 conjugated; red). Colocalizing viroplasms and RNAs are indicated by white arrows. Scale bar, 15 μm. (C) Quantitative analysis of NSP5-EGFP-positive structures (viroplasms) for RNA (gs6) in MA-NSP5-EGFP cells infected with rRV-wt or rRV-NSP5/S67A. ***, *P* < 0.001.

Having examined the viral protein composition of the aberrant viroplasms formed during infection with rRVs exhibiting impaired NSP5 phosphorylation, we then assessed their RNA content. Viral RNA transcripts were labeled by incorporation of 5-ethynyl uridine (5-EU) in actinomycin D-treated RV-infected cells, and total viral single-stranded RNA (ssRNA) was visualized by a reaction with Alexa Fluor 488 azide, as described in Materials and Methods. As expected, most viral transcripts localized in viroplasms of rRV-wt-infected cells (Fig. 6A), consistent with the roles of viroplasms in supporting viral replication and assembly. In contrast, no viral RNA transcripts could be detected in the aberrant structures in either wt MA104 or MA-NSP2-mCherry cells infected with the rRV-NSP5/S67A mutant (Fig. 6A). RNA accumulation in viroplasms was instead restored by infection with MA-NSP5 cells that supply wild-type NSP5 in *trans* (Fig. 6A, lower panel).

Although we did not detect RNA in aberrant structures (or viroplasms) in rRV-NSP5/S67A-infected MA104 cells by 5-EU staining, this mutant could still replicate (Fig. 3C). We then explored whether rRV-NSP5/S67A transcripts could accumulate in viroplasms, albeit with a much lower efficiency, i.e., beyond the sensitivity limit of 5-EU staining. For this purpose, we used single-molecule RNA fluorescence *in situ* hybridization (smFISH) to identify the sites of RV transcription. At 10 hpi, abundant gs6 transcripts could be detected in all viroplasms identified in MA-NSP5-EGFP cells infected with rRV-wt (Fig. 6B). Conversely, the rRV-NSP5/S67A-infected cells had sparse EGFP-tagged structures, but less than 20% of them contain gs6 RNA (Fig. 6B and C). Interestingly, the less frequently occurring rod-like aberrant structures also showed gs6 RNA accumulation, further suggesting that these structures could represent replication-functional organelles.

In contrast, smFISH performed on cells infected with rRV-NSP5/ΔT showed diffuse distribution of gs6 RNA that did not localize to any structures resembling viroplasms (Fig. 6B), also failing to support the virus genome replication (Fig. 4B).

**FIG 7:**
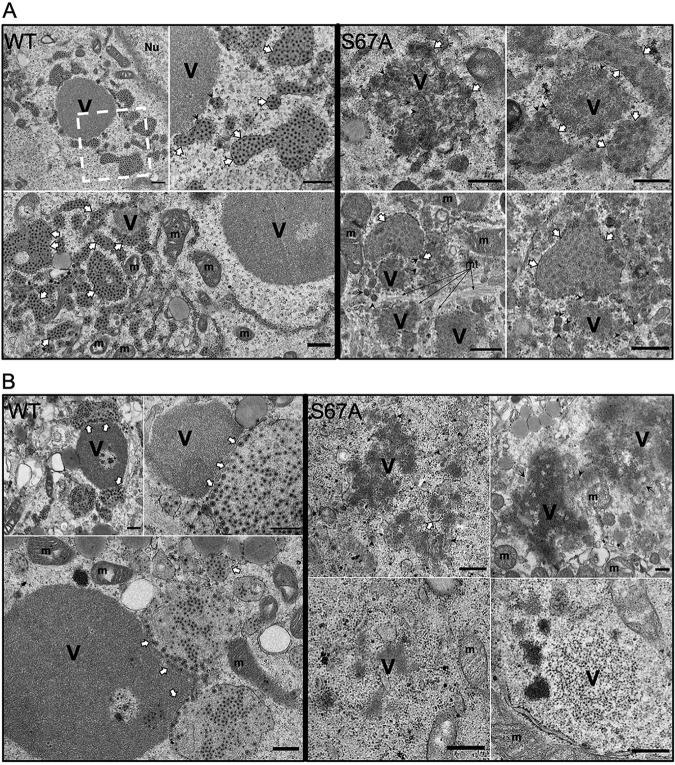
Electron microscopy of cells infected with rRV-NSP5/S67A. High-definition electron micrographs of MA104 (A) and MA-Δ3 (B) cells infected with rRV-wt (left panels) and rRV-NSP5/S67A (right panels) (MOI, 75 FFU/cell). At 10 hpi, cells were fixed with glutaraldehyde and processed for transmission electron microscopy. V, viroplasm; m, mitochondria; mt, microtubule bundles; Nu, nucleus. The white dashed box indicates the region that is shown magnified in the image to the immediate right. White arrows indicate the endoplasmic reticulum surrounding viroplasms; black arrowheads indicate viral particles with an envelope. Black arrows indicate microtubule bundles. Scale bar, 500 nm.

We then examined the ultrastructures of viroplasms with altered morphologies in the rRV-NSP5/S67A mutant by using electron microscopy. Upon infection with rRV-wt (Fig. 7A, left panel), multiple membraneless electron-dense inclusions encircled by the well-defined endoplasmic reticulum (ER) filled with triple-layered particles (TLPs) were present in the cells. At late infection points (10 hpi), ER filled with TLPs appeared to adopt a more tubular morphology, suggesting a successive step in the virus egress. In contrast, the rRV-NSP5/S67A-infected cells contained only a few immature viroplasms that lacked the ER network filled with TLPs (Fig. 7A, right panel). Only a few immature particles containing transient lipid membranes could be identified in cells infected with the rRV-NSP5/S67A mutant (Fig. 7A, right panel). Furthermore, the observed immature viroplasms also appeared to be less electron dense, likely due to their lower RNA composition and subsequently decreased number of available phosphate groups that bind contrasting UO^2+^ ions during the electron microscopy (EM) staining procedure. Together, these data strongly support the role of NSP5 phosphorylation in maintaining the viral RNA production and genome replication in viroplasms.

### Mechanism of NSP5 hyperphosphorylation.

We have previously proposed a model of the hierarchical NSP5 hyperphosphorylation associated with the assembly of viroplasms that involves a three-step mechanism: (i) an initial interaction of nonphosphorylated NSP5 with NSP2 (or VP2), (ii) phosphorylation of Ser67 by CK1α (this step does not take place when NSP5 is expressed alone), and (iii) hyperphosphorylation of NSP5 triggered by Ser67 phosphorylation that requires the 18-aa-long C-terminal tail (24).

**FIG 8:**
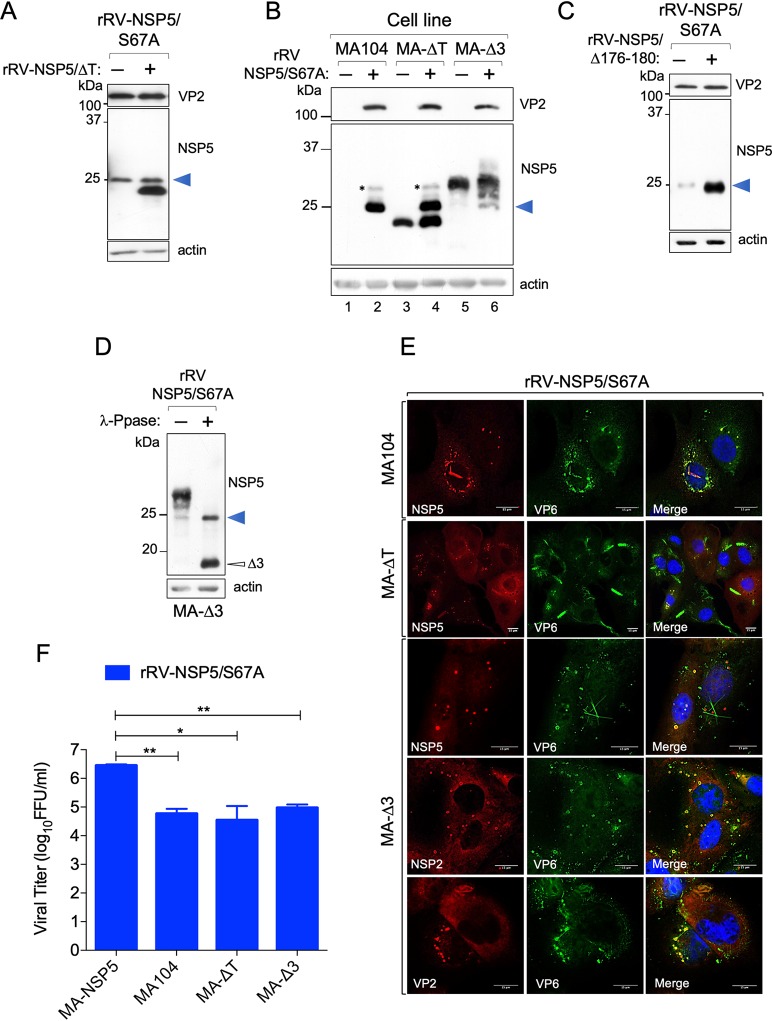
Phosphorylation of NSP5/S67A. (A) Western blot analysis of extracts of MA104 cells infected with rRV-NSP5/S67A or coinfected with rRV-NSP5/S67A and rRV-NSP5/ΔT. A blue arrowhead indicates NSP5/S67A. The faster-migrating band corresponds to NSP5-ΔT. (B) Western blot analysis of MA104, MA-ΔT, and MA-Δ3 cells infected with rRV-NSP5/S67A. A blue arrowhead indicates NSP5/S67A. (C) Western blot analysis of extracts of MA104 cells coinfected with rRV-NSP5/S67A and rRV-NSP5/Δ176-180. A blue arrowhead indicates NSP5/S67A. (D) λ-Ppase treatment of extracts of MA-Δ3 cells infected with the rRV-NSP5/S67A strain. Filled and open arrowheads indicate dephosphorylated NSP5-S67A and NSP5-Δ3, respectively. (E) Representative confocal immunofluorescence micrographs of MA104, MA-ΔT, and MA-Δ3 cells infected with rRV-NSP5/S67A. Cells were stained with the indicated antibodies. Scale bar, 15 μm. (F) Yield of infectious virus of rRV-NSP5/S67A grown in MA-NSP5, MA104, MA-ΔT, and MA-Δ3 cells at 24 hpi.*, *P* < 0.05; **, *P* < 0.01.

Here, we investigated the phosphorylation mechanism of NSP5 during RV infection using a number of NSP5 phosphorylation-negative rRV strains and MA104-derived stable transfectant cell lines (Table 1). We demonstrated that despite the presence of Ser67, deletion mutant NSP5/ΔT was not phosphorylated and failed to form viroplasms. We have previously shown that coexpression of NSP5/ΔT was also unable to trigger the phosphorylation cascade of NSP5/S67A, while other NSP5 mutants referred to here as activators of phosphorylation, e.g., NSP5/Δ3, did (24). Interestingly, upon coinfection with two rRVs, NSP5/S67A and NSP5/ΔT, neither of the mutated NSP5 variants were phosphorylated (Fig. 8A) (8, 19, 24, 26). This result was supported further by infecting the MA104 transfectant cell line stably expressing NSP5-ΔT (MA-ΔT) with the rRV-NSP5/S67A strain (Table 1 and Fig. 8B, lanes 3 and 4). The NSP5/Δ176-180 mutant was also unable to induce phosphorylation of NSP5/S67A following coinfection with the two rRVs, despite both containing Ser67 and the C-terminal tail. This result suggests that the “activator” NSP5 needs to also be hyperphosphorylated (Fig. 8C).

In MA-ΔT cells infected with the rRV-NSP5/S67A mutant strain, aberrant viroplasms similar to those observed in wt MA104 cells (Fig. 8E) were produced, which did not support virus replication compared to the wt virus (Fig. 8F).

In contrast to NSP5/ΔT, the NSP5 deletion mutant lacking amino acids 80 to 130 (NSP5/Δ3) becomes hyperphosphorylated when expressed alone and can function as an activator of NSP5/S67A phosphorylation (19, 24). We therefore asked whether an MA104 stable cell line expressing the deletion mutant NSP5/Δ3 (MA-Δ3) and infected with the rRV-NSP5/S67A strain was able to trigger hyperphosphorylation of NSP5/S67A and, as a consequence, sustain replication of the mutant rRV strain. As shown in Fig. 8B (lanes 5 and 6), NSP5/S67A was hyperphosphorylated in the presence of the NSP5/Δ3 mutant, which was confirmed by the λPpase treatment (Fig. 8D), although it did not completely rescue the phosphorylation pattern of NSP5 normally observed in rRV-wt infection. When loading large amounts of NSP5/S67A, we have occasionally observed a second faint band with reduced mobility, as seen in Fig. 8B, lane 2 (indicated with an asterisk). Interestingly, regular round structures resembling viroplasms, containing NSP5, NSP2, and VP2 with peripheral localization of VP6, were recovered in these cells infected with rRV-NSP5/S67A, and yet viral replication was nevertheless impaired (Fig. 8E and F). Consistently, the electron microscopy images showed structures of aberrant viroplasms similar to those obtained in MA104 wt cells (Fig. 7B, right panel).

**FIG 9:**
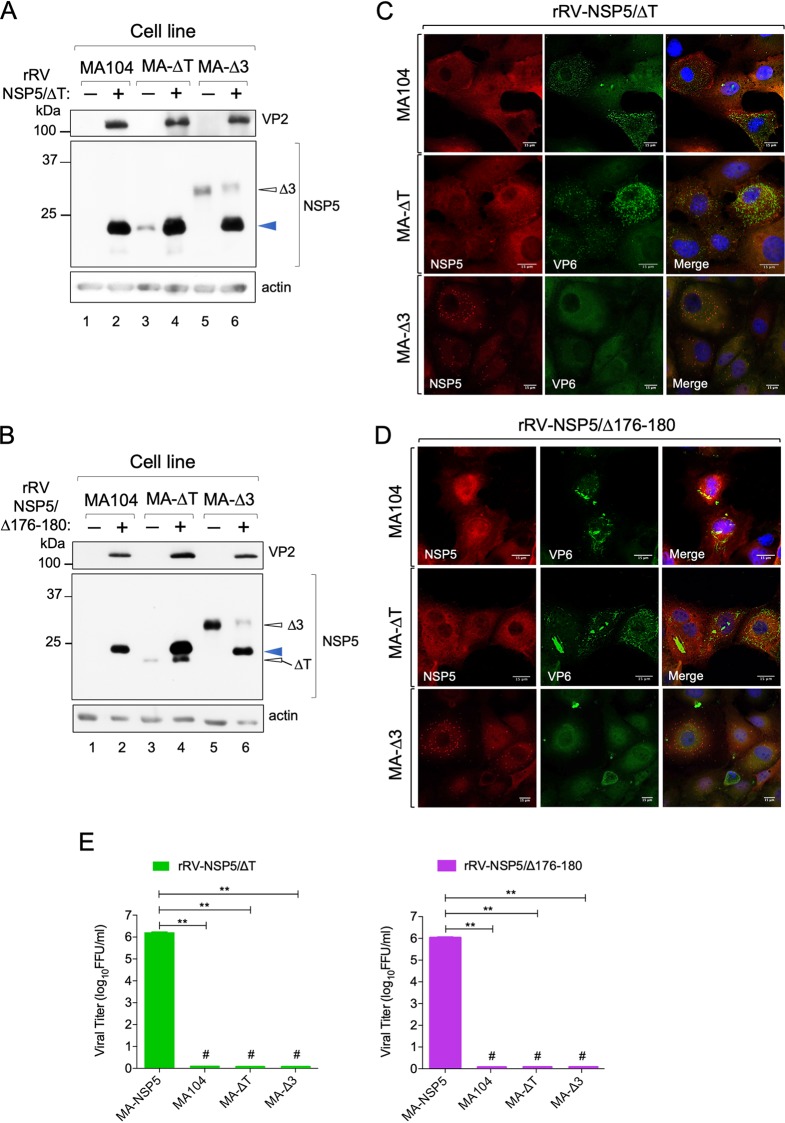
Phosphorylation of NSP5/ΔT and NSP5/Δ176-180 and viroplasm formation. Western blots (A and B) and confocal immunofluorescence micrographs (C and D) of MA104, MA-ΔT, and MA-Δ3 cells infected with rRV-NSP5/ΔT (A and C) or with rRV-NSP5/Δ176-180 (B and D). A blue arrowhead indicates NSP5/ΔT and NSP5/Δ176-180, respectively. Open arrowheads indicate NSP5-Δ3 and NSP5-ΔT. Cells were stained with the indicated antibodies and DAPI. Scale bar, 15 μm. (E) Single-step growth of rRV-NSP5/ΔT (left) and rRV-NSP5/Δ176-180 (right) in MA-NSP5, MA104, MA-ΔT, and MA-Δ3 cells, as indicated. The experiment was terminated at 24 hpi. **, *P* < 0.01; #, viral titer < 300 FFU/ml.

**FIG 10:**
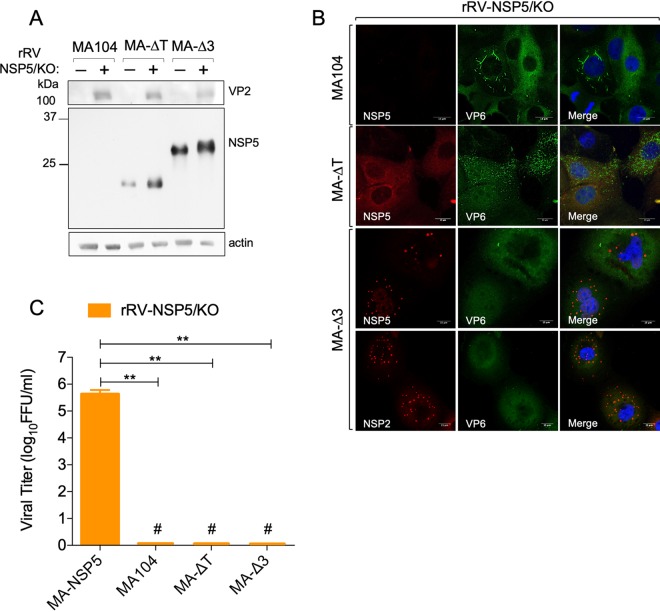
rRV-NSP5/KO in infected cells. Western blot analysis (A) and confocal immunofluorescence microscopy (B) of MA104, MA-ΔT, and MA-Δ3 cells infected with the rRV-NSP5/KO strain. Cells were stained with the indicated antibodies and DAPI. Scale bar, 15 μm. (C) Single-step growth of rRV-NSP5/KO in MA-NSP5, MA104, MA-ΔT, and MA-Δ3 cells, as indicated. The experiment was terminated at 24 hpi. **, *P* < 0.01; #, viral titer < 300 FFU/ml.

The NSP5 phosphorylation-negative mutants of the two other rRVs (NSP5/ΔT and NSP5/Δ176-180) did not undergo hyperphosphorylation in the MA-ΔT or MA-Δ3 cell lines (Fig. 9A and B). In both cases, viroplasm formation and virus replication were not rescued (Fig. 9C to E). Interestingly, apparently normal round structures were observed in MA-Δ3 cells, which, however, recruited significantly less VP6 (Fig. 9C and D). In addition, the previously observed VP6 spiky structures were not detected in these cells (Fig. 9C and D). Similar results were obtained with the rRV-NSP5/KO virus strain, which in MA-Δ3 cells did not replicate and also showed spherical structures that contained NSP5 and NSP2 but not VP6 (Fig. 10A to C).

Taken together, our data support a model of NSP5 hyperphosphorylation which absolutely requires the presence of the C-terminal tail and amino acid residues 176 to 180. Furthermore, NSP5 hyperphosphorylation requires Ser67 phosphorylation to initiate the phosphorylation cascade, thus playing a key role in the assembly of replication-competent viral factories.

## DISCUSSION

Rotaviruses replicate within cytoplasmic viral factories, or viroplasms. Most RV assembly intermediates, i.e., single-layered particles (cores) and double-layered particles (DLPs), are concentrated primarily in viroplasms. Following budding of DLPs into the lumen of the endoplasmic reticulum, the immature particles acquire a transient envelope, as well as the outer capsid proteins VP4 and VP7, resulting in a mature triple-layered virion. Moreover, downregulation of expression of the most abundant viroplasm-forming proteins, NSP5 and NSP2, severely impacts the formation of viroplasms and production of virus progeny (13, 15, 25, 31). In light of these observations, viroplasms have long been recognized as essential compartments supporting RV replication.

Of the two nonstructural proteins involved in viroplasm assembly, NSP5 appears to play a crucial role by potentially providing a scaffold that allows for recruitment of additional viral proteins. Only when NSP5 is coexpressed with NSP2 and/or VP2 do these proteins assemble into the viroplasm-like structures (VLS), which are also capable of recruiting additional structural proteins, including VP1, VP2, and VP6 (8, 10). Given these observations, we hypothesized that complete removal of NSP5 would be lethal for RV replication. Using a modified reverse-genetics system for rotaviruses (29), we here provide the first direct evidence of the essential role of NSP5 in viroplasm formation and viral replication. In order to characterize replication-deficient NSP5-negative mutants, we have established a *trans*-complementing system that provides NSP5 to the virus both transiently in BHK-T7 cells and stably in the NSP5-producing MA104 cell line (MA-NSP5), thus enabling facile isolation of rRVs lacking functional NSP5. Using this approach, we have demonstrated that NSP5-deficient rRV was unable to form viroplasms and replicate in the wt MA104 cells, while the viroplasm formation and viral replication were efficiently rescued in the *trans*-complementing MA-NSP5 cell line. Interestingly, the rRVs generated using this method also failed to incorporate dsRNA originating from the NSP5-encoding mRNA lacking the 5′ and 3′ untranslated regions (UTRs), further suggesting the essential roles of UTRs for genome packaging in RVs (35, 36).

NSP5 hyperphosphorylation has been previously implicated in the regulation of NSP5 assembly into viroplasms. This phosphorylation, however, requires the interaction of NSP5 with either NSP2 or VP2, as NSP5 is not phosphorylated when expressed alone (8, 22, 23). Previous studies have suggested that activation of NSP5 hyperphosphorylation may require a conformational change that leads to its efficient hyperphosphorylation via a positive-feedback loop mechanism (8, 23, 24). Two regions comprising the N-terminal amino acids 1 to 33 (region 1) and the central region amino acids 81 to 130 (region 3) have been reported to prevent NSP5 phosphorylation in the absence of other viral proteins, while the 18-aa-long C-terminal tail was found to be essential for its phosphorylation (8, 23, 24).

Here, we have shown that all three rRV mutants, rRV-NSP5/S67A, rRV-NSP5/ΔT, and rRV-NSP5/Δ176-180, expressing the phosphorylation-negative NSP5 variants, were unable to form round viroplasms upon infection of MA104 cells. Interestingly, further analysis of these mutants reveals some key differences between each NSP5 variant. While the rRV-NSP5/S67A strain formed aberrant structures resembling viroplasms that poorly support RV replication, this variant was still capable of producing infectious progeny, in contrast to the two other rRV mutant strains. Interestingly, the phenotype observed with the NSP5/S67A mutant was essentially the same as the one previously reported with the wt virus infecting MA104 cells silenced for expression of CK1α, which is involved in phosphorylating Ser67 and initiating the hyperphosphorylation cascade (24, 25). It has recently been shown that CK1α is also involved in phosphorylating NSP2, controlling the formation of rotavirus viral factories (37). Our data obtained with the rRV-NSP5/S67A mutant strongly suggest that the lack of NSP5 hyperphosphorylation determines both the morphogenesis of viroplasms and their capacity to support RV genome replication. We cannot rule out the role of NSP2 phosphorylation in the assembly of viroplasms, since NSP2 is also likely to be phosphorylated by CK1α upon infection of the rRV-NSP5/S67A strain. Despite the formation of aberrant structures resembling viroplasms, the amount of RNA produced within those structures in rRV-NSP5/S67A-infected MA104 cells was practically below the detection limit of 5-EU labeling, while the RNA replication was fully rescued in the *trans*-complementing MA-NSP5 cell line (Fig. 5). It is unlikely that the viral mRNAs produced in MA104 wt cells infected with the rRV-NSP5/S67A strain were degraded faster, as most of the ssRNA synthesized is a consequence of the secondary round of transcription from the newly made dsRNA-containing particles. Indeed, very small amounts of dsRNA were detected during the infection of MA104 cells. smFISH results confirmed the presence of small amounts of plus ssRNA in some of these aberrant structures. This result is consistent with the finding that the rRV-NSP5/S67A strain did replicate, albeit at much lower levels than rRV-wt. Thus, these structures are likely to sustain virus replication with decreased efficiency, which was further confirmed by the electron microscopy analysis of MA104 cells infected with the rRV-NSP5/S67A strain.

The important role of NSP5 hyperphosphorylation was further supported by the results obtained with the two phosphorylation-negative mutant strains, NSP5/ΔT and NSP5/Δ176-180, which possess Ser67 and yet failed to form viroplasms in MA104 cells. Surprisingly, the Δ176-180 mutant, containing the C-terminal tail, also failed to form viroplasms in MA104 cells, despite the absence of Ser or Thr within the chosen aa 176-to-180 region. Both phosphorylation-negative mutants tested did not replicate in MA104 cells, and we could not detect any structures containing viral RNA.

Using the NSP5 mutant strains described above and the established stable transfectant MA104 cells, we were able to investigate the molecular mechanism that leads to NSP5 hyperphosphorylation. We showed that the NSP5/S67A mutant from the rRV was indeed hyperphosphorylated, albeit not completely, when infecting MA-Δ3 cells, restoring the round morphology of the structures resembling viroplasms with a complete absence of the aberrant structures observed in MA104 cells. This finding strongly suggests that impairment of NSP5 phosphorylation is the direct cause of the formation of the aberrant structures in the cytosol of the infected cell. Despite the fact that these structures appeared morphologically similar to the classical round viroplasms in MA-Δ3 cells, the presence of VP6 around these structures was observed only with the hyperphosphorylated NSP5/S67A mutant, not with the other NSP5 phosphorylation-negative mutants. One possibility is that during RV infection, accumulation of VP6 in round-shaped viroplasms requires a full-length hyperphosphorylated NSP5, as well as phosphorylation of multiple serine residues, likely by CK2 (17, 26). Moreover, the round structures found in MA-Δ3 cells infected with rRV-NSP5/KO failed to contain VP6.

The observed failure to rescue the replication of the rRV-NSP5/S67A strain to the wt levels in MA-Δ3 cells could be the consequence of the incomplete recovery of the complex pattern of phosphorylated isoforms of wt NSP5. This suggests that some intermediate isoforms might be important for the formation of fully functional replication-competent viroplasms.

**FIG 11:**
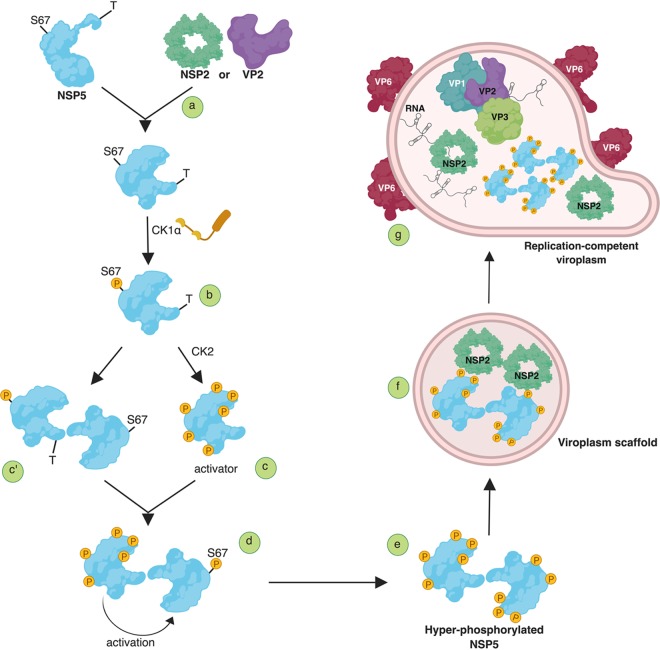
Model of NSP5 hyperphosphorylation and assembly of replication-competent viroplasms. (a) Interaction of nonphosphorylated NSP5 with either NSP2 or VP2 is required to (b) induce conformational changes that make S67 available for CK1α phosphorylation (P). This initial step of the cascade is then (c) sequentially completed by phosphorylation by CK2 of other residues, including serines in domain 4, to generate the NSP5 activator or (c′) a step of interaction with a nonphosphorylated molecule precedes the involvement of CK2. (d) NSP5 interactions to form dimers/multimers are mediated by the carboxy-terminal tails (T). (e) The primed (S67-phosphorylated) activator molecules result in substrate activation and fully hyperphosphorylated NSP5. (f) Assembly of viroplasm scaffolds containing NSP2 serve to (g) recruit the other viroplasmic components, VP1, VP2, VP3, and VP6 to assemble replication-competent viroplasms. P, phosphorylated amino acid. (This image was created using BioRender.)

We propose a model of the complex hierarchical mechanism of NSP5 hyperphosphorylation during RV infection (Fig. 11). It involves the interaction of NSP5 with either NSP2 or VP2 (Fig. 11a), required to make Ser67 available for CK1α phosphorylation (Fig. 11b). This initial step is then sequentially completed by CK2-mediated phosphorylation of other serines to generate the NSP5 “activator” (Fig. 11c) during a step dependent on the interaction of the Ser67-phosphorylated molecules with the nonphosphorylated partners in the NSP5 oligomeric complexes (Fig. 11d). Alternatively, oligomers could be formed before the activation step (Fig. 11c′). NSP5 interactions mediated by the carboxy-terminal tails T result in substrate activation and a fully hyperphosphorylated NSP5 (Fig. 11e). This process leads to the assembly of viroplasm scaffolds containing NSP2 (Fig. 11f) and recruitment of VP6, as well as the other viroplasmic proteins, VP1, VP2, and VP3, to assemble replication-competent viroplasms (Fig. 11g).

The hierarchical phosphorylation of proteins appears to be a common mechanism regulating many cellular processes. In mammalian cells, hierarchical phosphorylation has been described for β-catenin, in which Ser45 phosphorylated by CK1α primes it for hyperphosphorylation by glycogen synthase kinase 3 (GSK-3) (38, 39), which triggers its ubiquitination and proteasomal degradation (40). A similar phosphorylation mechanism has recently been described for nonstructural protein NS5A of the hepatitis C virus (HCV), with the hyperphosphorylation cascade primed by the initial phosphorylation of serine 225 by CK1α and the subsequent phosphorylation of neighboring residues involving other kinases (41, 42). Moreover, NS5A phosphorylation has been shown to play a key role in controlling the establishment of replication complexes during HCV infection (43). Similarly, a number of other nonstructural viral proteins have been shown to undergo multiple phosphorylation events during virus infection, suggesting that these complex posttranslational modifications play a pivotal role in orchestrating the assembly of replication-competent viral factories (44–46).

## MATERIALS AND METHODS

### Cells and viruses.

MA104 (embryonic African green monkey kidney cells, ATCC CRL-2378.1, from Chlorocebus aethiops), U2OS (human bone osteosarcoma epithelial cells), Caco-2 (colorectal adenocarcinoma human intestinal epithelial cell line, ATCC HTB-37), and HEK293T (human embryonic kidney epithelial cells, ATCC CRL-3216) cells were cultured in Dulbecco’s modified Eagle’s medium (DMEM) (Life Technologies) supplemented with 10% fetal bovine serum (FBS) (Life Technologies) and 50 μg/ml gentamicin (Biochrom AG).

MA104-NSP5-EGFP (MA-NSP5-EGFP) cells (7) were cultured in DMEM supplemented with 10% FBS (Life Technologies), 50 μg/ml gentamicin (Biochrom AG), and 1 mg/ml Geneticin (Gibco-BRL, Life Technologies).

MA104-NSP2-mCherry (MA-NSP2-mCherry), MA104-Δ3 (MA-Δ3), MA104-Δtail (MA-ΔT), and MA104-NSP5wt (MA-NSP5) stable transfectant cell lines (embryonic African green monkey kidney cells, ATCC CRL-2378) were grown in DMEM (Life Technologies) containing 10% FBS, 50 μg/ml gentamicin (Biochrom AG), and 5 μg/ml puromycin (Sigma-Aldrich).

BHK-T7 cells (baby hamster kidney stably expressing T7 RNA polymerase) were cultured in Glasgow medium supplemented with 5% FBS, 10% tryptose phosphate broth (TPB) (Sigma-Aldrich), 50 μg/ml gentamicin (Biochrom AG), 2% nonessential amino acids (NEAA), and 1% glutamine.

Recombinant simian RV strain SA11 (rRV-wt), rescued using the reverse-genetics system using cDNA clones encoding wild-type SA11 (G3P[2]) virus (28), was propagated in MA104 cells cultured in DMEM supplemented with 0.5 μg/ml trypsin (Sigma-Aldrich).

### Recombinant RV titration.

Recombinant NSP5 mutant RVs were grown in MA-NSP5 cells, and the lysate was serially diluted 2-fold and used to infect MA-NSP5 cells, seeded in 24-well plates with coverslips. After 1 h of adsorption, virus was removed, and cells were incubated at 37°C. At 5 h postinfection (hpi), cells were fixed with 4% paraformaldehyde (PFA) in phosphate-buffered saline (PBS) (137 mM NaCl, 2.7 mM KCl, 8.1 mM Na_2_HPO_4_, and 1.74 mM KH_2_PO_4_ [pH 7.5]) for 15 min at room temperature and permeabilized for 5 min with PBS containing 0.01% Triton X-100. Next, cells were incubated for 30 min with PBS supplemented with 1% bovine serum albumin (PBS-BSA) at room temperature and then with anti-NSP5 (1:1,000) (22) or anti-VP2 (1:200) (7, 22) or anti-NSP2 (1:200) (7, 22) guinea pig serum diluted in PBS-BSA. After washing three times with PBS, cells were incubated for 1 h at room temperature with TRITC (tetramethyl rhodamine isocyanate)-conjugated anti-guinea pig IgG (Jackson ImmunoResearch) (1:500) diluted in PBS-BSA.

Nuclei were stained with ProLong diamond antifade mountant with DAPI (4′,6-diamidino-2-phenylindole) (Thermo Scientific). Samples were imaged using a confocal setup (Zeiss Airyscan equipped with a 63× objective with a numerical aperture [NA] of 1.3). Each viroplasm-containing cell was counted as one focus-forming unit (FFU). The average number of cells with viroplasms of six fields of view per each virus dilution was determined, and the total number of cells containing viroplasms in the whole preparation was estimated. The virus titer was determined as:

$$\text{virus titer}\left(\frac{\text{FFU}}{\text{ml}}\right)=\frac{N\times \text{dilution factor}}{V\left(\text{ml}\right)}$$

where *N* is a total number of cells containing 1 or more viroplasms and *V* is the volume of virus inoculum added.

### Replication kinetics of recombinant viruses.

MA104 cell line (ATCC CRL-2378.1) or stably transfected MA104 cells (NSP5; Δ3, ΔT) were seeded into 24-well plates and subsequently infected with recombinant RVs at a multiplicity of infection (MOI) (FFU/cell) of 0.5 for multistep growth curve experiments and an MOI of 5 for a single-step growth curve experiment. After adsorption for 1 h at 37°C, the cells were washed twice with PBS and the medium was replaced with DMEM without trypsin. After incubation at 37°C, the cells were harvested after 8, 16, 24, and 36 h of virus adsorption. The cell lysates were freeze-thawed three times and activated with trypsin (1 μg/ml) for 30 min at 37°C. The lysates were used to infect monolayers of MA-NSP5 cells seeded in a μ-Slide 8-well chamber slide (iBidi GmbH, Munich, Germany). The cells were then fixed 5 h postinfection for 15 min with 4% paraformaldehyde and permeabilized for 5 min with PBS containing 0.01% Triton X-100. Next, cells were incubated for 30 min with PBS-BSA at room temperature and then with anti-NSP5 serum (1:1,000) diluted in PBS-BSA (22). After three washes with PBS, cells were incubated for 1 h at room temperature with TRITC-conjugated anti-guinea pig IgG (Jackson ImmunoResearch) (1:500) diluted in PBS containing 1% BSA (PBS-BSA).

The number of infected cells was counted, and the virus titers were expressed in focus-forming units per ml (FFU/ml).

### Plasmid construction.

RV plasmids pT_7_-VP1-SA11, pT_7_-VP2-SA11, pT_7_-VP3-SA11, pT_7_-VP4-SA11, pT_7_-VP6-SA11, pT_7_-VP7-SA11, pT_7_-NSP1-SA11, pT_7_-NSP2-SA11, pT_7_-NSP3-SA11, pT_7_-NSP4-SA11, and pT_7_-NSP5-SA11 (28) were used to rescue recombinant RVs by reverse genetics. pT_7_-NSP5/S67A carrying a T220G nucleotide mutation in gs11 and pT_7_-NSP5/Tyr18Stop harboring a T75G nucleotide substitution were generated by QuikChange II site-directed mutagenesis (Agilent Technologies) from pT_7_-NSP5-SA11.

pT_7_-NSP5/ΔT was generated from pT_7_-NSP5-SA11 by deleting the last 18 C-terminal amino acids (FALRMRMKQVAMQLIEDL) using substitution of the F181-encoding triplet with a stop codon. pT_7_-NSP5/Δ176-180 was obtained by deleting amino acids 176 to 180 (YKKKY). The described deletions were performed using the QuikChange II site-directed mutagenesis kit (Agilent Technologies).

pcDNA3-NSP5 and pcDNA3-NSP2 used for the rescue of recombinant rotaviruses were obtained as previously described (8).

For the generation of lentiviral plasmids, NSP5 and NSP2-mCherry were amplified by PCR from pT_7_-NSP5-SA11 (28) and pCI-NSP2-mCherry (47), respectively, and inserted into plasmid pAIP (Addgene no. 74171 [48]) at the NotI-EcoRI restriction enzymes sites to yield pAIP-NSP5 and pAIP-NSP2-mCherry. NSP5/ΔT was amplified from the pT_7_-NSP5/ΔT by PCR and inserted into pPB-MCS (Vector Builder) at the NheI-BamHI sites to generate pPB-NSP5/ΔT.

pPB-NSP5/Δ3 was generated with a GenParts DNA fragment (GenScript) containing NSP5 ORF lacking amino acids 80 to 130 (VKTNADAGVSMDSSAQSRPSSNVGCDQVDFSLNKGLKVKANLDSSISIST) and inserted into the NheI-BamHI sites of pPB-MCS vector. The sequences of the NSP5 mutants have been deposited in GenBank (see below).

### Generation of stable cell lines.

MA-NSP5-EGFP cells were generated as previously described (7).

MA-NSP2-mCherry and MA-NSP5 cell lines were generated using a lentiviral vector system (49). Briefly, HEK293T cells were maintained in DMEM (Life Technologies) supplemented with 10% FBS (Life Technologies) and 50 μg/ml gentamicin (Biochrom AG). Approximately 7 × 10^6^ HEK293T cells were seeded in 10-cm^2^ tissue culture dishes 24 h before transfection. For each well, 2.4 μg of pMD2-VSV-G, 4 μg of pMDLg pRRE, 1.8 μg of pRSV-Rev, and 1.5 μg of plasmid containing pAIP-NSP2-mCherry or pAIP-NSP5 and the human immunodeficiency virus long terminal repeats were cotransfected with Lipofectamine 3000 (Sigma-Aldrich) according to the manufacturer’s instructions. After 48 h, the virus was collected by filtration with a 0.45-μm polyvinylidene fluoride filter and was immediately used or stored at −80°C. For lentiviral transduction, MA104 cells were transduced in six-well plates with 1 ml of lentiviral supernatant for 2 days.

MA-Δ3 and MA-ΔT were generated using the PiggyBac technology (50). Briefly, 10^5^ MA104 cells were transfected with the pCMV-HyPBase (50) and transposon plasmids pPB-NSP5/Δ3 and pPB-NSP5/ΔT using a ratio of 1:2.5 with Lipofectamine 3000 (Sigma-Aldrich) according to the manufacturer’s instructions.

The cells were maintained in DMEM supplemented with 10% FBS for 3 days, and then the cells were incubated with DMEM supplemented with 10% FBS and 5 μg/ml puromycin (Sigma-Aldrich) for 4 days to allow the selection of cells expressing the gene of interest.

### Rescue of rRVs from cloned cDNAs.

To rescue recombinant RV (rRV) strain SA11 (rRV-wt), monolayers of BHK-T7 cells (4 × 10^5^) cultured in 12-well plates were cotransfected using 2.5 μl of TransIT-LT1 transfection reagent (Mirus) per μg of DNA plasmid. Each mixture comprised 0.8 μg of the SA11 rescue plasmids pT_7_-VP1, pT_7_-VP2, pT_7_-VP3, pT_7_-VP4, pT_7_-VP6, pT_7_-VP7, pT_7_-NSP1, pT_7_-NSP3, and pT_7_-NSP4 and 2.4 μg of pT_7_-NSP2 and pT_7_-NSP5 (29). Furthermore, 0.8 μg of pcDNA3-NSP2 and 0.8 μg of pcDNA3-NSP5, encoding NSP2 and NSP5 proteins, were also cotransfected to increase rescue efficiency.

To rescue rRVs encoding NSP5 mutants, pT_7_ plasmids encoding NSP5/S67A, NSP5/Y18Stop, and NSP5/Δ180-198 segments were used instead of pT_7_-NSP5. At 24 h posttransfection, MA-NSP5 cells (5 × 10^4^ cells) were added to transfected cells to provide a functional NSP5 for the virus rescue. The cells were cocultured for 3 days in FBS-free medium supplemented with trypsin (0.5 μg/ml) (Sigma-Aldrich). After incubation, transfected cells were lysed by freeze-thawing, and 200 μl of the lysate was transferred to fresh MA-NSP5 cells. After adsorption at 37°C for 1 h, the cells were washed three times with PBS and further cultured at 37°C for 4 days in FBS-free DMEM supplemented with 0.5 μg/ml trypsin (Sigma-Aldrich no. 9002-07-7) until a clear cytopathic effect was visible. The recombinant viruses were sequenced to rule out the presence of unexpected mutations or reversions.

### Immunofluorescence microscopy.

Immunofluorescence experiments were performed using a μ-Slide 8-well chamber slide (iBidi GmbH, Munich, Germany) and the following antibody dilutions: anti-NSP5 guinea pig serum, 1:1,000; anti-NSP2 guinea pig serum, 1:200; anti-VP2 guinea pig serum, 1:500 (7, 16, 22); anti-VP6 mouse monoclonal antibody, 1:1,000 (clone 4B2D2) (generously provided by J. L. Zambrano and F. Liprandi, Instituto Venezolano de Investigaciones Científicas, Caracas, Venezuela); Alexa Fluor 488-conjugated anti-mouse antibody, 1:500 (Life Technologies); and TRITC-conjugated anti-guinea pig antibody, 1:500 (Life Technologies).

### 5-EU labeling.

Newly synthesized RNAs were labeled by including 2 mM 5-ethynyl uridine (5-EU) into the cell culture medium, and modified incorporated nucleotides were reacted with an azide-conjugated fluorophore, Alexa Fluor 488, by following the manufacturer’s protocol for the Click-iT RNA Alexa Fluor 488 imaging kit (Thermo Fisher Scientific). Cell nuclei were stained with ProLong diamond antifade mountant with 4′,6-diamidino-2-phenylindole (DAPI; Thermo Scientific). Samples were imaged using a confocal setup (Zeiss Airyscan equipped with a 63× objective with an NA of 1.3), and the images were processed using ZEN lite software.

### RNA FISH.

Rotavirus-infected MA104 cells were fixed with 4% (vol/vol) paraformaldehyde in nuclease-free Dulbecco’s phosphate saline buffer (DPBS) for 10 min at room temperature. Samples were washed twice with DPBS and then permeabilized with 70% (vol/vol) ethanol in RNase-free water at +4°C for at least 1 h prior to hybridization. Permeabilized samples were rehydrated for 5 min in a prehybridization buffer (300 mM NaCl, 30 mM trisodium citrate [pH 7.0] in nuclease-free water, 10% [vol/vol] formamide, supplemented with 2 mM vanadyl ribonucleoside complex). Rehydrated samples were hybridized with a 62.5 nM concentration of an equimolar mixture of Cy3-labeled DNA probes designed to target the coding region of gene segment 6 of simian rotavirus A/SA11 (GenBank accession no. AY187029.1) using Stellaris Probe Designer v2 software (LCG Biosearch Technologies), in a total volume of 200 μl of the hybridization buffer (Stellaris RNA fluorescence *in situ* hybridization [FISH] hybridization buffer, SMF-HB1-10 [Biosearch Technologies], supplemented with 10% [vol/vol] deionized formamide). After 4 to 8 h of incubation at 37°C in a humidified chamber, samples were briefly rinsed with the wash buffer (300 mM NaCl, 30 mM trisodium citrate, pH 7.0, 10% [vol/vol] formamide in nuclease-free water), after which a fresh aliquot of 300 μl of the wash buffer was applied to each sample and incubated twice at 37°C for 30 min. After 2 washes, nuclei were briefly stained with 300 nM DAPI solution in 300 mM NaCl, 30 mM trisodium citrate, pH 7.0, and the samples were finally rinsed with and stored in the same buffer without DAPI prior to imaging.

### Transmission electron microscopy.

MA104 cells were seeded at 1 × 10^5^ cells in 2-cm^2^ wells onto sapphire discs and infected at an MOI of 75 FFU/cell. At 10 hpi, cells were fixed with 2.5% glutaraldehyde in 100 mM Na/K phosphate buffer, pH 7.4, for 1 h at 4˚C and kept in that buffer overnight at 4˚C. Afterward, samples were postfixed with 1% osmium tetroxide in 100 mM Na/K phosphate buffer for 1 h at 4˚C and dehydrated in a graded ethanol series starting at 70%, followed by two changes in acetone, and embedded in Epon. Ultrathin sections (60 to 80 nm) were cut and stained with uranyl acetate and lead citrate (47). Samples were analyzed in a transmission electron microscope (CM12; Philips, Eindhoven, The Netherlands) equipped with a charge-coupled-device (CCD) camera (Ultrascan 1000; Gatan, Pleasanton, CA, USA) at an acceleration of 100 kV.

### λ-Ppase and CIP assays.

Cellular extracts were incubated with 2,000 U of λ-protein phosphatase (λ-Ppase) (New England Biolabs) in 50 mM Tris-HCl (pH 7.5), 0.1 mM EDTA, 5 mM dithiothreitol (DTT), 0.01% Brij 35, and 2 mM MnCl_2_. The mixture was incubated at 30°C for 2 h. Samples were loaded into an SDS-PAGE gel and analyzed by Western blotting.

For the CIP assay, cellular extracts were incubated with 1,000 U of calf intestinal alkaline phosphatase (CIP) (New England Biolabs) in CutSmart reaction buffer (New England Biolabs) and incubated at 30°C for 2 h. Samples were subjected to SDS-PAGE and analyzed by Western blotting.

### Electrophoresis of viral dsRNA genomes.

Cells were infected with the recombinant viruses at an MOI of 5 and lysed 16 h postinfection. Total RNA was extracted from lysed cells with RNAzol (Sigma-Aldrich) according to the manufacturer’s protocol, and the dsRNA segments were resolved on 10% (wt/vol) PAGE gels for 2 h at 180 V and visualized by ethidium bromide staining (1 μg/ml).

### Protein analysis.

Proteins derived from rRV-infected cellular extract were separated on an SDS-PAGE gel for 2 h at 35 mA and transferred to polyvinylidene difluoride membranes (Millipore; IPVH00010) for 1 h 30 min at 300 mA (24). For protein analysis, membranes were incubated with the following primary antibodies: anti-NSP5 (1:5,000) (16), anti-VP2 (1:5,000) (10), and anti-NSP2 guinea pig sera (1:2,000). The membranes were then incubated with the corresponding horseradish peroxidase (HRP)-conjugated goat anti-guinea pig sera (1:10,000; Jackson ImmunoResearch). Mouse HRP-conjugated anti-actin monoclonal antibody (MAb) (1:35,000) (clone AC-15; Sigma-Aldrich) was used as a loading control.

Signals were detected using the enhanced chemiluminescence system (Pierce ECL Western blotting substrate; Thermo Scientific).

### Statistics used.

Statistical analysis and plotting were performed using GraphPad Prism 6 software (GraphPad Prism 6.0; GraphPad Software Inc., La Jolla, CA, USA). Error bars represent standard deviations. Data were considered to be statistically significant when a *P* of <0.05 was determined by Student's *t* test.

### Data availability.

Sequences for NSP5/S67A, NSP5/ΔT, NSP5/Δ176-180, NSP5/Δ3, and NSP5/KO are available in GenBank under accession numbers MN520458, MN520459, MN520460, MN520461, and MN520462, respectively.
